# c-di-GMP and AHL signals-triggered chemical communication under electrical signaling disruption restores *Geobacter sulfurreducens* biofilm formation

**DOI:** 10.1093/ismeco/ycae096

**Published:** 2024-07-20

**Authors:** Qian Zhu, Yanyan Zheng, Xingwang Zhou, Dunjia Wang, Mengjiao Yuan, Dingkang Qian, Sha Liang, Wenbo Yu, Jiakuan Yang, Huijie Hou, Jingping Hu

**Affiliations:** School of Environmental Science and Engineering, Huazhong University of Science and Technology, 1037 Luoyu Road, Wuhan 430074, Hubei,, China; College of Chemistry and Chemical Engineering, Hubei Key Laboratory of Pollutant Analysis and Reuse Technology, Hubei Normal University, 11 Cihu Road, Huangshi 435002, Hubei, China; College of Chemistry and Chemical Engineering, Hubei Key Laboratory of Pollutant Analysis and Reuse Technology, Hubei Normal University, 11 Cihu Road, Huangshi 435002, Hubei, China; College of Chemistry and Chemical Engineering, Hubei Key Laboratory of Pollutant Analysis and Reuse Technology, Hubei Normal University, 11 Cihu Road, Huangshi 435002, Hubei, China; College of Chemistry and Chemical Engineering, Hubei Key Laboratory of Pollutant Analysis and Reuse Technology, Hubei Normal University, 11 Cihu Road, Huangshi 435002, Hubei, China; School of Environmental Science and Engineering, Huazhong University of Science and Technology, 1037 Luoyu Road, Wuhan 430074, Hubei,, China; Hubei Provincial Engineering Laboratory of Solid Waste Treatment, Disposal and Recycling, 1037 Luoyu Road, Wuhan 430074, Hubei, China; School of Environmental Science and Engineering, Huazhong University of Science and Technology, 1037 Luoyu Road, Wuhan 430074, Hubei,, China; Hubei Provincial Engineering Laboratory of Solid Waste Treatment, Disposal and Recycling, 1037 Luoyu Road, Wuhan 430074, Hubei, China; School of Environmental Science and Engineering, Huazhong University of Science and Technology, 1037 Luoyu Road, Wuhan 430074, Hubei,, China; Hubei Provincial Engineering Laboratory of Solid Waste Treatment, Disposal and Recycling, 1037 Luoyu Road, Wuhan 430074, Hubei, China; School of Environmental Science and Engineering, Huazhong University of Science and Technology, 1037 Luoyu Road, Wuhan 430074, Hubei,, China; Hubei Provincial Engineering Laboratory of Solid Waste Treatment, Disposal and Recycling, 1037 Luoyu Road, Wuhan 430074, Hubei, China; School of Environmental Science and Engineering, Huazhong University of Science and Technology, 1037 Luoyu Road, Wuhan 430074, Hubei,, China; Hubei Provincial Engineering Laboratory of Solid Waste Treatment, Disposal and Recycling, 1037 Luoyu Road, Wuhan 430074, Hubei, China; State Key Laboratory of Coal Combustion, Huazhong University of Science and Technology, 1037 Luoyu Road, Wuhan 430074, Hubei, China; School of Environmental Science and Engineering, Huazhong University of Science and Technology, 1037 Luoyu Road, Wuhan 430074, Hubei,, China; Hubei Provincial Engineering Laboratory of Solid Waste Treatment, Disposal and Recycling, 1037 Luoyu Road, Wuhan 430074, Hubei, China; School of Environmental Science and Engineering, Huazhong University of Science and Technology, 1037 Luoyu Road, Wuhan 430074, Hubei,, China; Hubei Provincial Engineering Laboratory of Solid Waste Treatment, Disposal and Recycling, 1037 Luoyu Road, Wuhan 430074, Hubei, China; State Key Laboratory of Coal Combustion, Huazhong University of Science and Technology, 1037 Luoyu Road, Wuhan 430074, Hubei, China

**Keywords:** electrical signaling, chemical communication, electrogenic biofilm, c-di-GMP, quorum sensing, bioelectrochemical system

## Abstract

Electrogenic biofilms, which have attracted considerable attention in simultaneous wastewater treatment and energy recovery in bioelectrochemical systems, are regulated by chemical communication and potassium channel-mediated electrical signaling. However, how these two communication pathways interact with each other has not been thoroughly investigated. This study first explored the roles of chemical communication, including intracellular bis-(3′-5′)-cyclic dimeric guanosine monophosphate (c-di-GMP) and extracellular N-acyl-homoserine lactone (AHL)-mediated quorum sensing, in electrogenic biofilm formation through an integrated analysis of transcriptomics and metabolomics. Electrical signaling disruption inhibited the formation and electroactivity of *Geobacter sulfurreducens* biofilm, which was mainly ascribed to the reduction in biofilm viability and extracellular protein/polysaccharide ratio. The upregulation of expression levels of genes encoding c-di-GMP and AHL synthesis by transcriptomic analysis, and the increased secretion of N-butanoyl-L-homoserine lactone by metabolomic analysis confirmed the enhancement of chemical communication under electrical signaling disruption, thus indicating a compensatory mechanism among different signaling pathways. Furthermore, protein–protein interaction network showed the convergence of different signaling pathways, with c-di-GMP-related genes acting as central bridges. This study highlights the interaction of different signaling pathways, especially the resilience of c-di-GMP signaling to adverse external stresses, thereby laying the foundation for facilitating electrogenic biofilm formation under adverse conditions in practical applications.

## Graphical Abstract

**Graphical Abstract:**
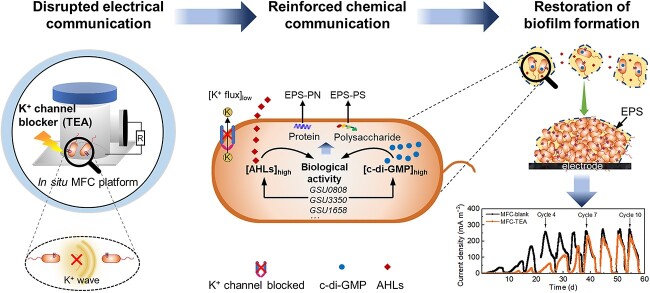

## Introduction

Electrogenic biofilms, which possess unique capabilities of extracellular electron transfer (EET), have attracted considerable interest in bioelectrochemical system (BESs) for wastewater treatment, energy conversion, biosensing, bioremediation, etc., [1, 2]. Besides, like non-electrogenic biofilms, electrogenic biofilms in the aggregated state are also more resistant to environmental inhibitors than planktonic bacteria, which is highly beneficial for bacteria to overcome the vulnerability to adverse conditions [3, 4]. Notably, the cells in electrogenic biofilm require electrical or chemical signals to coordinate population-level behaviors, which directly influences biofilm formation, EET efficiency and adaptive responses to adverse external factors [5–7]. Therefore, the regulation of electrogenic biofilm formation from the perspective of signal communication has been recognized as a feasible strategy for optimizing BES performance [8, 9].

N-acyl-homoserine lactones (AHLs)-mediated quorum sensing has been widely studied for cell-to-cell signal communication in electrogenic biofilms, which can be used to induce biofilm formation [8], increase the relative abundance of exoelectrogen *Geobacter* [10], enhance pollutant removal [11, 12], etc. In addition, bis-(3′-5′)-cyclic dimeric guanosine monophosphate (c-di-GMP), as an intracellular secondary messenger, has recently been reported to regulate multicellular aggregation especially under adverse environmental stresses in nonelectrochemical systems, where high intracellular c-di-GMP level will promote the secretion of extracellular polymeric substances (EPS), thereby stimulating biofilm formation [13, 14]. Anammox bacteria used intracellular c-di-GMP to sense and relay diverse adverse factors (e.g. low pH, aerobic condition, and low temperature) and further modulate allosteric alterations of c-di-GMP effectors for granule formation [3]. Notably, the studies on c-di-GMP signaling in electrogenic biofilms are very limited.

In addition to chemical communication (c-di-GMP and AHLs), electrical signaling mediated by potassium channels, in the form of spatially propagating waves of potassium, has recently been revealed to play critical roles in biofilm formation [6, 15]. Electrical signaling disruption by blocking potassium channels or knocking out the key gene *gsuK* significantly deteriorated the formation of *Geobacter sulfurreducens* biofilm [7, 16]. Therefore, both chemical communication and potassium channel-mediated electrical communication are responsible for electrogenic biofilm formation. However, is there a convergence between chemical and electrical communication? How do they interact with each other? These have not been thoroughly studied in electrogenic biofilms so far.

The interactions between quorum sensing and c-di-GMP signaling have been studied in several non-BESs, but there is no universal mechanism [17]. Quorum sensing and c-di-GMP signaling reciprocally controlled the biofilm formation of *Vibrio cholerae*, with extracellular quorum sensing autoinducers sensing information about vicinal bacterial community and intracellular c-di-GMP sensing environmental information within cells [18]. Another study implied that quorum sensing negatively affected cellular c-di-GMP production in *Pseudomonas aeruginosa* [19]. Further, AHLs and c-di-GMP regulons have been reported to overlap for the regulation of 24 genes in *Burkholderia cenocepacia* H111, with high c-di-GMP level inhibiting the expression of AHL-related genes [20]. Notably, studies on the interactions between electrical signaling and chemical communication have been scarcely reported for both electrogenic and non-electrogenic biofilms. Specifically, the interactions among different signal communication pathways have not been well studied so far and still remain an open question, especially for electrogenic biofilms.

Herein, a single-chambered microbial fuel cell (MFC) platform inoculated with pure *G. sulfurreducens* were constructed to observe the changes in EPS compositions and electrogenic biofilm formation induced by electrical signaling disruption in situ. Besides, the effects of electrical signaling disruption on c-di-GMP-related and AHL-related gene expressions were analyzed using transcriptomics and PPI networks. Moreover, the production of chemical signals under electrical signaling disruption were further investigated using non-targeted metabolomics. This study aims to lay theoretical foundations for regulating electrogenic biofilm formation and adaptability to adverse conditions from a perspective of signal communication.

## Materials and methods

### Microbe culturing and MFC operation

*G. sulfurreducens* was cultured in modified acetate-fumarate medium as previously reported [21], with 10 mM sodium acetate as the electron donor and 50 mM sodium fumarate as the electron acceptor. All fresh medium was aerated with 20% CO_2_ and 80% N_2_ for 60 min to completely remove oxygen and then autoclaved at 121°C for 20 min before use.

Single-chambered, air-cathode MFCs (18 ml in volume) was utilized for in situ observation of the formation of *G. sulfurreducens* biofilm using confocal laser scanning microscopy (CLSM, TCS SP8, Leica, Germany) (See online supplementary material for a colour version of Fig. S1). Indium tin oxide (ITO) with 5 cm × 5 cm × 1.1 mm was used as the anode, and a membrane electrode assembly was used as the cathode, which was fabricated by hot-pressing a proton exchange membrane (Nafion 117, Dupont) onto a traditional air-cathode with a diameter of 10 mm [22]. External resistor of 10 Ω was connected between the ITO anode and membrane electrode assembly to record the voltage output. Prior to use, the *in-situ* MFCs were sterilized with 75% ethanol and then illuminated with ultraviolet light for 60 min.

Log-phase cultures of *G. sulfurreducens* were harvested by centrifugation (5500 g, 4°C, 10 min), washed using deoxygenated sodium fumarate-free fresh medium and resuspended in the above fresh medium to an OD_600_ of 0.2. Then the inoculum was added to each MFC chamber, and tetraethylammonium (TEA), a non-selective potassium channel blocker, was also added with a final concentration of 20 mM to inhibit the electrical signaling, designated as MFC–TEA. Control group (defined as MFC-blank) that received no TEA was also fabricated for comparison. The inoculum was only added at the first cycle, while TEA was added throughout the experiments. Each group had at least three replicates, and all MFCs were run under fed-batch mode (i.e., the chambers were refilled with fresh medium when the current density decreased to <5 mA m^−2^ forming one complete cycle of operation) at 28°C.

### Electrochemical activity, biofilm viability, and EPS composition analysis

A data acquisition instrument (Keithley 2750, Tektronix, USA) was utilized to record the voltage output across the resistor (10 Ω) of all MFCs every 10 min. Non-turnover cyclic voltammetry (CV) and non-turnover differential pulse voltammetry (DPV) were conducted using ITO anode as working electrode, a Ag/AgCl (saturated KCl solution, 0.197 V *vs* SHE) as reference electrode and membrane electrode assembly as counter electrode, and the parameters were set based on previous literature [22].

To detect the biofilm viability, the bulk solution in MFCs was first drawn off using an injection syringe, then the chambers were washed with sterile 0.9% NaCl solution and injected with dyes from a LIVE/ DEAD BacLight Bacterial Viability Kit (Thermo Fisher Scientific Inc., USA), and the anodic biofilms were stained in the dark for 30 min. Afterwards, the anodic biofilms were washed with sterile 0.9% NaCl solution to remove excess dyes, and then observed by CLSM. Finally, deoxygenated sodium fumarate-free fresh medium was injected into the MFC reactors to continue the culturing of anodic biofilms.

To detect the compositions of EPS, the bulk solution in MFCs was first drawn off using an injection syringe, then the chambers were washed with sterile 0.9% NaCl solution. The anodic biofilms were then stained with fluorescein isothiocyanate (FITC) and tetramethylrhodamine-concanavalin A (Con A) sequentially to detect the proteins and α-polysaccharides in the EPS, respectively. The detailed staining procedure, the corresponding excitation and emission wavelengths of FITC and Con A are described in previously published literature [23].

### Extraction of biofilm RNA and transcriptomic analysis

After six cycles of operation, the anodic biofilms were scraped using sterile cell scrapers and placed in sterilized 50 mM phosphate buffer solution, and the above samples were then centrifuged at 5500 g for 15 min to collect the *G. sulfurreducens* cells and immediately frozen in liquid nitrogen. Afterwards, Trizol Reagent (Thermo Fisher Scientific, USA) was utilized to extract total RNA from the above biofilm samples. RNase-Free DNase I (Takara Inc., Japan) was used to eliminate residual DNA. RNA degradation and contamination was monitored on 1% agarose gels, and its integrity was measured using the RNA Nano 6000 Assay Kit of the Bioanalyzer 2100 system (Agilent Technologies, CA, USA). The mRNA was then enriched by removing the rRNA using Ribo-zero kit (Epicentre Inc., USA), and randomly fragmented into short fragments by adding fragmentation buffer, and then utilized as the templates for cDNA synthesis as described previously [24]. Subsequently, the cDNA was purified using AMPure XP beads (Beckman Coulter, Beverly, USA), followed by PCR amplification [22]. Finally, the PCR products were purified, followed by the evaluation of library quality using Agilent Bioanalyzer 2100, and the paired-end sequencing was performed by Illumina Novaseq platform. All the above procedures were carried out by Novogene Bioinformatics Technology Co., Ltd. (Tianjin, China). Raw data has been submitted to NCBI database with accession numbers of SRR21146804 and SRR21146805.

Kyoto Encyclopedia of Genes and Genome (KEGG) and Gene ontology (GO) databases were used to determine the enriched pathways. PPI analysis of multiple signal communication pathways was carried out using STRING database (https://string-db.org/), and the results were visualized by importing them into Cytoscape software (version 3.9.0).

### Untargeted metabolomic analysis

At the end of the fourth, fifth, and sixth cycles of MFCs, the bulk solutions in the chambers were collected and centrifuged at 5500 g for 15 min to obtain the supernatant, which was first frozen at −20°C for 2 days and then freeze-dried using a freeze dryer. The lyophilized powders from the fourth, fifth, and sixth cycles from the same reactor were combined, weighed and sent to Majorbio Bio-Pharm Technology Co., Ltd. (Shanghai, China) for subsequent metabolite determination. Briefly, 20 mg of each sample was accurately weighed and 400 μl of extract solution (V_methanol_: V_water_ = 4:1, containing 0.02 mg/ml of L-2-chlorophenylalanine as internal standard) was added for metabolite extraction. The samples were then centrifuged for 15 min (13 000 g, 4°C) and the supernatant obtained was transferred to an injection vial for analysis by liquid chromatography-mass spectrometer (UHPLC-Triple TOF, AB SCIEX, USA). At the same time, 20 μl of supernatant was pipetted from each sample separately, mixed and used as quality control samples. Details of chromatographic and mass spectrometric conditions can be found in Text S1 in Supplementary Materials.

### Statistical analysis

The significance of differences was determined using a Student’s two-tailed *t*-test (^*^P < 0.05, ^*^^*^P < 0.01 and ^*^^*^^*^P < 0.001). Partial least squares-discriminant analysis (PLS-DA) model were utilized to evaluate the overall difference in metabolite profile between MFC-TEA and MFC-blank, and 200 times random permutation tests were used to verify the validity and reliability of the model. Besides, variable importance in the projection (VIP) value in the PLS-DA model could be utilized to indicate the contribution of each metabolite to the discriminations between MFC-TEA and MFC-blank. The differential metabolites were selected based on VIP > 1.5 and *P* value < 0.05.

## Results and discussion

### Electrogenic biofilm formation in response to electrical signaling disruption

**Fig. 1:**
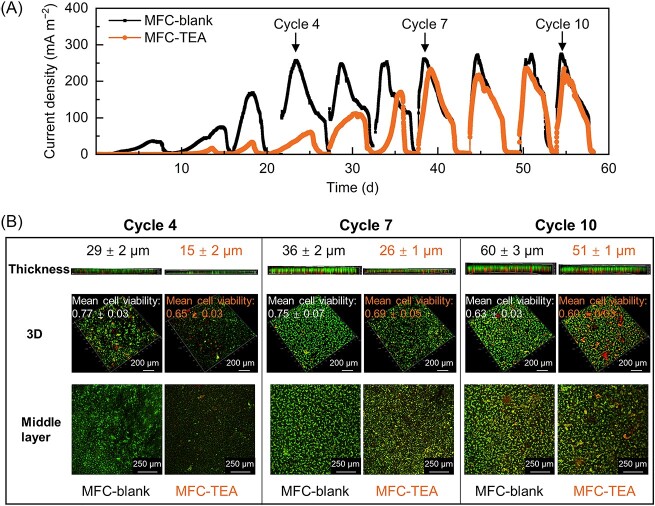
(A) Representative output current density diagram of MFC-blank and MFC-TEA, which was selected from three independent reactors. (B) Representative CLSM images of biofilms in MFC-blank and MFC-TEA at cycle 4, 7 and 10 . Biofilm thickness was calculated based on the height of the fluorescence-containing positions in the CLSM 3D image, as CLSM forms a 3D image of the sample by scanning it layer by layer; the mean cell viability (i.e., ratio of live to total cells) was calculated by dividing the area of live cells by the area of total cells using Leica Application Suite software (Leica, Germany); the middle layer referred to the layer that was in the middle of the 3D image. The standard deviations of biofilm thickness and mean cell viability were calculated from CLSM images of three independent reactors (biological replicates).

The addition of potassium channel blocker (TEA) adversely affected *G. sulfurreducens* biofilm formation and electricity generation performance. As shown in Fig. 1A, the current density gradually increased in MFC-blank during the first cycle of the operation, suggesting that *G. sulfurreducens* was effectively growing onto the ITO anode, whereas the current density barely increased in MFC-TEA, indicating that TEA addition exerted a detrimental influence on cell activity or microbial attachment onto the anode. Besides, MFC-blank achieved a maximum current density of 266.6 ± 10.4 mA m^−2^ on Day 23 (during the 4th cycle), while MFC-TEA reached 231.1 ± 9.4 mA m^−2^ on Day 39 (during the 7th cycle). This indicated that TEA addition decelerated *G. sulfurreducens* biofilm formation, which might be ascribed to the deterioration of bacterial agglomeration under cell-to-cell electrical signaling disruption [25].

Biofilm monitoring using CLSM imaging (green for live cells and red for dead cells) further confirmed that TEA addition decreased biofilm viability and further decelerated *G. sulfurreducens* biofilm formation (Fig. 1B). At Cycle 4, the biofilm formed under TEA stress was sparse, with a thickness of 15 ± 2 μm, significantly lower than the 29 ± 2 μm of the control (*P* < 0.01), indicating that biofilm growth was inhibited by TEA addition. Besides, the ratio of live to dead cells was 0.65 ± 0.03, significantly lower than 0.77 ± 0.03 of the control (*P* < 0.05), further indicating that TEA addition decreased cell viability. At Cycle 7, the biofilm in MFC-TEA was able to grow uniformly onto the ITO anode and the mean cell viability increased to 0.69 ± 0.05, indicating an adaptive response of electrogenic biofilms to TEA-induced communication stress. At Cycle 10, the mean cell viability decreased to 0.63 ± 0.03 and 0.60 ± 0.03 in MFC-blank and MFC-TEA, respectively, probably due to the thick biofilms (60 ± 3 and 51 ± 1 μm, respectively) with the accumulation of dead cells in the inner biofilm layer [26]. Overall, TEA-induced electrical signaling disruption adversely affected biofilm formation and viability.

Furthermore, TEA addition decreased the ratio of extracellular proteins to extracellular polysaccharides (PN/PS) in EPS compared with the control (See online supplementary material for a colour version of Fig. S2). At Cycle 4, the EPS components were unevenly distributed onto the ITO anode in MFC-TEA, which was consistent with the sparse distribution of biofilm in Fig. 1B. Besides, the ratio of PN/PS in MFC-TEA was 1.15 ± 0.10, which was lower than that in MFC-blank. At Cycle 7, the ratio of PN/PS increased to 1.89 ± 0.14 and 1.53 ± 0.11 in MFC-blank and MFC-TEA, respectively. The proteins in EPS, particularly cytochrome c, have been reported to mediate the electron transfer from the cells to extracellular solid electron acceptors, whereas polysaccharides with inferior conductivity may interfere with electron transfer [27]. The significant increase in PN/PS ratios at Cycle 7 indicated a high electroactivity of electrogenic biofilms in both MFCs. At Cycle 10, the decreased PN/PS ratios in both MFCs indicated that electrogenic biofilms secreted more polysaccharides for cell-protection, which was consistent with the decrease in biofilm viability (as shown in Fig. 1B) [23]. The secretion of polysaccharide is usually stimulated by unfavorable environmental stresses as an adaptive response, thus more polysaccharide production in MFC-TEA compared with MFC-blank was probably due to signal communication stress (i.e. electrical signaling disruption) in this study. Overall, MFC-TEA showed increased secretion of polysaccharides at each stage compared to MFC-blank, indicating that electrical signaling disruption exerted communication stress on *G. sulfurreducens* cells.

### Biofilm electroactivity in response to electrical signaling disruption

**Fig. 2:**
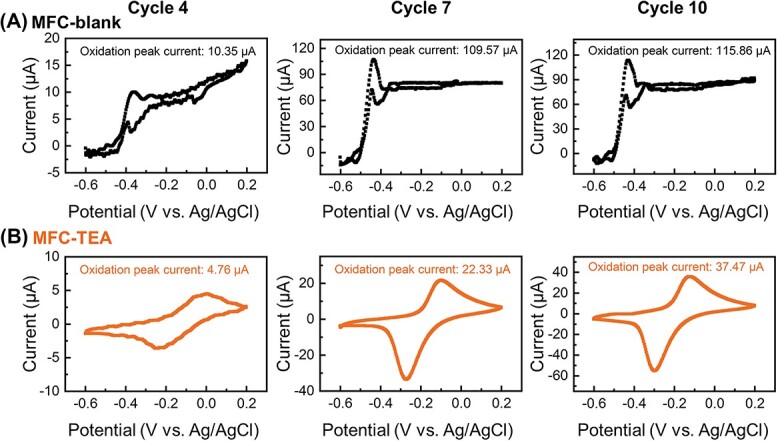
Representative CV curves of anodic biofilms under non-turnover conditions in (A) MFC-blank and (B) MFC-TEA (the scan rate was set at 5 mV s^−1^, and the scan range was set at −0.6 ~ 0.2 V).

Electrical signaling disruption decreased the electroactivity of *G. sulfurreducens* biofilm. As shown in Fig. 2A and B*G. sulfurreducens* biofilm achieved lower catalytic currents than the control under TEA stress, with oxidation peak current of 4.76, 22.33, and 37.47 μA at Cycle 4, 7, and 10, respectively. Besides, TEA addition shifted the potential of major oxidation peak in a positive direction compared to the control, i.e. from −0.37 V (control) to −0.02 V at Cycle 4; from −0.44 V (control) to −0.10 V at Cycle 7; from −0.43 V (control) to −0.12 V at Cycle 10. Since the potential of redox peak represents the redox-active component, particularly cytochromes [10], the changes in potential indicated that electrical signaling disruption might have altered the redox-active components or contents [28]. It calls for special attention that there existed some differences in the shapes of CV curves between MFC-blank and MFC-TEA, with a large potential difference between oxidation and reduction peak potentials for MFC-TEA, indicating an inferior biofilm electroactivity under TEA stress [10, 29]. This was further confirmed by DPV results (See online supplementary material for a colour version of Fig. S3). Notably, the biofilm electroactivity in MFC-TEA increased with operation time, suggesting that *G. sulfurreducens* cells might have made adaptive responses to TEA-induced communication stress.

### Signal communication-related gene regulation in response to electrical signaling disruption

Transcriptomic profiling of anodic biofilms was performed at Cycle 6 to investigate how *G. sulfurreducens* cells responded adaptively to TEA-induced electrical signaling disruption. As shown in GO enrichment analysis in, See online supplementary material for a colour version of, Fig. S4A, some transmembrane transport processes and ATP-related processes had more upregulated genes than downregulated genes under electrical signaling disruption (MFC-TEA *vs*. MFC-blank), indicating an active metabolic state of the electrogenic biofilms in MFC-TEA, which was consistent with the successful growth of anodic biofilm at this stage (Fig. 1). As shown in KEGG analysis in, See online supplementary material for a colour version of, Fig. S4B, two-component system (gsu02020), bacterial chemotaxis (gsu02030), and ABC transporters (gsu02010) were significantly enriched (Padj <0.05). Two-component systems are important pathways for microbes to sense, transmit external signals and regulate gene expressions in response to environmental stimuli, and it globally regulates almost all physiological activities of bacterial cells [28]. Besides, certain two-component systems are closely associated with intracellular c-di-GMP signaling and extracellular AHL-mediated quorum sensing [1, 30]. The significant enrichment of two-component systems (involving 23 differentially expressed genes) under TEA stress suggested that TEA could be sensed by two-component system, which then regulated gene expressions to enhance microbial adaption to adverse conditions. ABC transporters can derive energy from ATP cleavage for the transport of a variety of substances such as amino acids, vitamins and nucleotides, for both nutrition and signal communication [31, 32], and its enrichment indicated that microbial metabolic and signaling processes might be active, thereby facilitating biofilm formation.

**Fig. 3:**
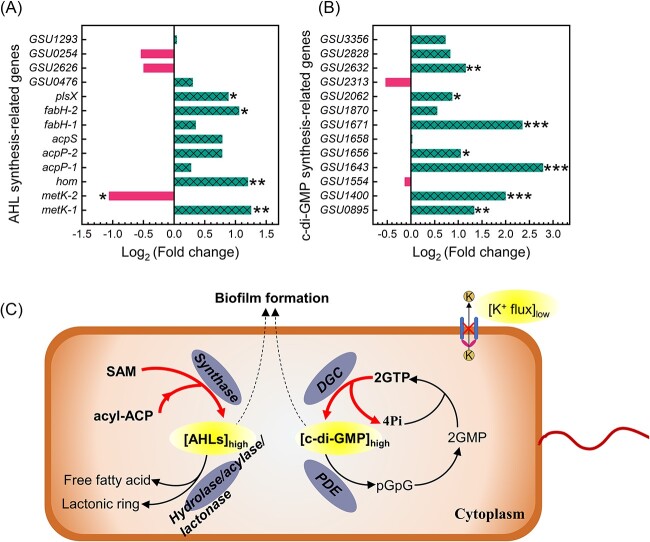
Transcriptomic analysis of functional genes encoding (A) AHL synthesis and (B) c-di-GMP synthesis in anodic biofilms at the end of cycle 6. Log_2_ (fold change) indicates the differential expression values of genes in MFC-TEA *vs*. MFC-blank, and a positive value of Log_2_(fold change) indicates a higher expression level in MFC-TEA than in MFC-blank, while a negative value denotes a lower expression level in MFC-TEA. Asterisks indicate the statistical significance of difference in MFC-TEA *vs*. MFC-blank, as indicated below: ^*^p < 0.05, ^*^^*^p < 0.01 and ^*^^*^^*^p < 0.001. (C) Illustration of the reinforcement of c-di-GMP signaling and AHL-mediated quorum sensing for biofilm formation in response to the disruption of potassium channel-mediated electrical signaling. Transcriptomic samples were obtained from the combined anodic biofilms of three independent reactors (biological replicates).

*G. sulfurreducens* cells regulated the expressions of chemical communication-related genes in response to electrical signaling disruption. As shown in Fig. 3A and Table S1, most genes encoding AHL synthesis were upregulated, especially *metK-1* (S-adenosylmethionine synthetase, *GSU1880*), *hom* (Homoserine dehydrogenase, *GSU1693*), *fabH-2* (3-oxoacyl-(acyl carrier protein) synthase III, *GSU1601*), and *plsX* (Fatty acid synthesis protein, *GSU1600*) with significance (*P* < 0.05). Two substrates, S-adenosyl-L-methionine (SAM) and acyl-acyl carrier protein (acyl-ACP), are required for the synthesis of AHLs [33, 34], and the upregulation of *metK-1* gene (encoding S-adenosylmethionine synthetase) can support the synthesis of SAM. The *hom* gene has been reported to play important roles in the synthesis of AHLs, with *hom*-deficient strains producing lower levels of AHLs than wild strains [35]. In addition, the upregulation of *fabH-2* and *plsX* genes contributed to fatty acid metabolism and thus to the synthesis of acyl-ACP and SAM [33]. Furthermore, most genes encoding AHL degradation were downregulated, especially *GSU3451* (alpha/beta hydrolase family), *GSU1378* (Serine hydrolase) and *GSU2450* (alpha/beta hydrolase family) (*P* < 0.01) (See online supplementary material for a colour version of Fig. S5A). The downregulation of these genes further contributed to the accumulation of AHLs.

Furthermore, the expressions of genes encoding c-di-GMP synthesis and degradation indicated an enhancement of c-di-GMP signaling under electrical signaling disruption. The synthesis and degradation of intracellular c-di-GMP are regulated by diguanylate cyclase and phosphodiesterase, respectively, with diguanylate cyclase synthesizing c-di-GMP via the GGDEF domain and phosphodiesterase hydrolyzing c-di-GMP via the HD-GYP or EAL domain [14]. As shown in Fig. 3B, most genes encoding c-di-GMP synthesis were significantly upregulated, especially *GSU1671* (response receiver-modulated diguanylate cyclase, GGDEF family), *GSU1643* (response receiver-modulated diguanylate cyclase, GGDEF family), *GSU1400* (sensor diguanylate cyclase, GGDEF family), *GSU2632* (diguanylate cyclase, GGDEF family), *GSU0895* (sensor diguanylate cyclase, GGDEF family), *GSU2062* (diguanylate cyclase, GGDEF family) and *GSU1656* (response receiver sensor diguanylate cyclase, GGDEF family) (*P* < 0.05), with *GSU1643* having the highest Fold change value of 6.9. With regard to the genes encoding c-di-GMP degradation, significant downregulation was observed in *GSU0699* (cyclic diguanylate phosphodiesterase, HD family), *GSU2511* (EAL domain-containing protein, EAL family), *GSU1037* (EAL domain-containing protein, EAL family) and *GSU0537* (EAL domain-containing protein, EAL family) (*P* < 0.001), with *GSU0537* having the highest Fold change value of 4.6 (See online supplementary material for a colour version of Fig. S5B). The downregulation of these genes further contributed to the accumulation of c-di-GMP. Taken together, electrical signaling disruption induced the reinforcement of c-di-GMP signaling and AHL-mediated quorum sensing for biofilm formation (Fig. 3C).

Notably, the number of c-di-GMP-related genes with significant upregulation or downregulation was higher than that of AHL-related genes, and the Fold change values of most c-di-GMP-related genes were also greater, indicating that c-di-GMP signaling might be more responsive and imperative to TEA stress than quorum sensing. Intracellular c-di-GMP, as a critical bacterial secondary messenger, can specifically interact with versatile downstream effectors, thus regulating diverse physiological processes including polysaccharide secretion, stress resistance and biofilm formation [36]. Previous research has showed that the combination of *Rhizobium* sp. NJUST18 and *Shinella granuli* NJUST29 had both strong pyridine degradation and aggregation abilities due to the high content of intracellular c-di-GMP which mediated EPS secretion at both transcriptional and posttranslational levels [37]. Thus, the lower PN/PS ratio in MFC-TEA (See online supplementary material for a colour version of Fig. S2) was possibly associated with intracellular c-di-GMP signaling, which promoted the secretion of extracellular polysaccharides to facilitate the adaption of electrogenic biofilms to TEA-induced unfavorable external conditions.

The interactions between AHLs-mediated quorum sensing and c-di-GMP signaling have been studied in several non-electroactive biofilms, but there is no universal mechanism. AHLs in *Nitrospira* spp. (common microbes in activated sludge community) have been reported to indirectly regulate multiple downstream genes via the c-di-GMP pathway [38]. Quorum sensing negatively influenced c-di-GMP production in *P. aeruginosa* [19]. The interactions among different signal communication pathways (AHLs, c-di-GMP, electrical signaling, etc.), whether in electroactive biofilms, on other surfaces or even in similar nature conditions, remain to be thoroughly investigated.

**Fig. 4:**
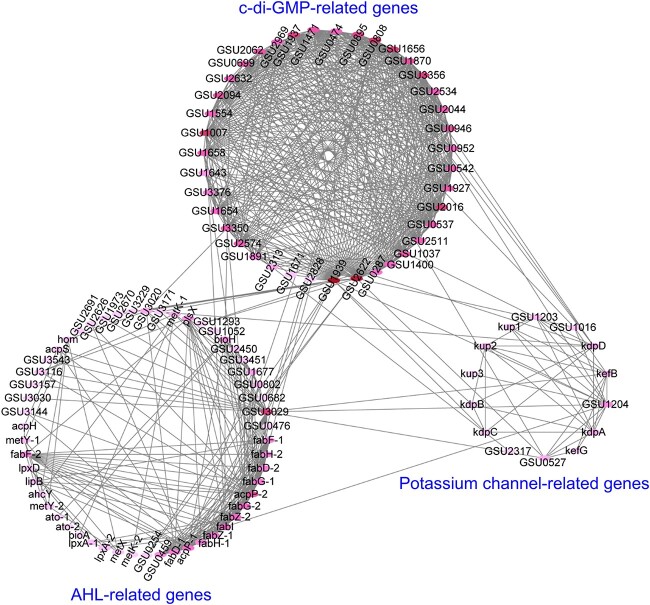
PPI analysis among the genes encoding potassium channels, c-di-GMP and AHLs in *G. sulfurreducens* based on STRING database. The genes associated with SAM, acyl-ACP, acyl-homoserine-lactone hydrolase/acylase/lactonase have been grouped in AHLs; the genes associated with diguanylate cyclase and phosphodiesterase have been grouped in c-di-GMP.

PPI network can be used to systematically analyze the interactions of a wide range of proteins (or genes) in a microbial system in order to explore how proteins (or genes) work and to further uncover core functional genes for subsequent precise regulation [22]. As shown in Fig. 4, the depth of red color on each node indicates the degree of interaction of the gene (represented by the node) with other genes, with darker color indicating stronger interaction with other genes. The prediction of numerous and close direct interactions among c-di-GMP genes suggests that c-di-GMP signaling may play significant roles in signal communication of *G. sulfurreducens* biofilm, which was consistent with previous research showing that c-di-GMP signaling had crucial roles in EPS secretion and flagella regulation of *G. anodireducens* biofilm under electric field [1]. Notably, there are fewer edges between potassium channel-related genes and AHL-related genes, suggesting their weak direct interactions, with intracellular c-di-GMP signaling probably acting as a bridge for their linking. Specifically, the genes *GSU0808*/*GSU3350*/*GSU1658* (all encoding c-di-GMP synthesis), *GSU2622*/*GSU1939* (both encoding c-di-GMP degradation) link both AHL-related and potassium channel-related genes (Fig. 4), indicating their potentially critical roles in the coordination of multiple signal communication pathways. The *fabH*, *bioA* and *acpP* genes associated with AHLs were reported to significantly down-regulated (*P* < 0.01) in *Geobacter soli* biofilms grown with addition of acylase (a kind of quorum quenching enzyme) *vs*. control [8]. A potassium channel (KdpA), ATPase (KdpB), and a secondary membrane component thought to mediate affinity (KdpC) have been reported to form a high-affinity transporter component in a variety of bacterial cells [39]. The *GSU0808* gene was reported to be significantly up-regulated in *G. sulfurreducens* cells grown on poised electrodes at 0.5 mA compared with cells grown with Fe(III)-citrate as the electron acceptor [40]. Therefore, further investigations of the roles of these key genes through genetic engineering (knockout or overexpression) will provide a deeper understanding of the interactions among multiple signal communication pathways, which may open up new ideas for rapid biofilm formation as well as regulation of biofilm properties.

### Metabolic pathway analysis in response to electrical signaling disruption

**Fig. 5:**
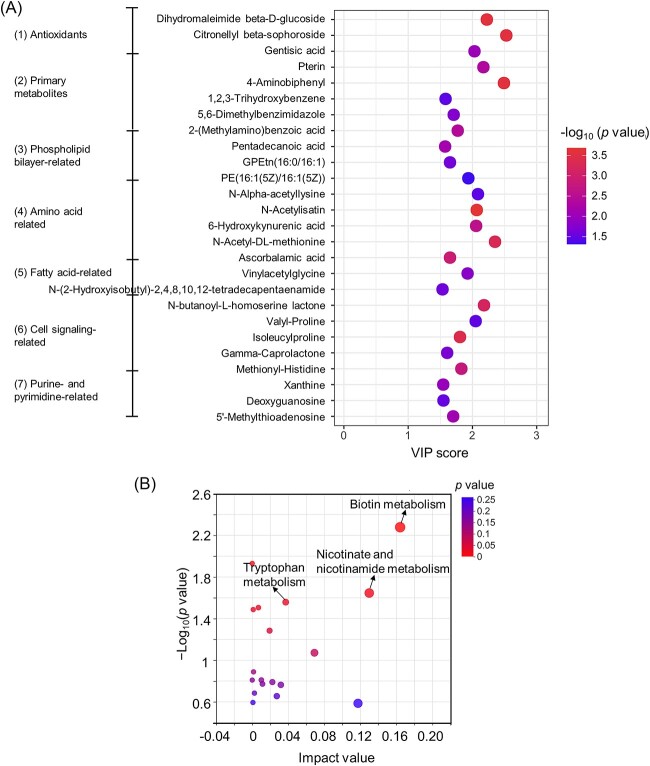
(A) Upregulated differential metabolites and their VIP scores used to rank their contribution to the difference in MFC-TEA *vs*. MFC-blank. (B) KEGG pathway enrichment analysis of differential metabolites in MFC-TEA *vs*. MFC-blank (note: Each bubble represents a KEGG pathway, and the horizontal and vertical axes indicate the impact value and statistical significance of the pathway, respectively. The bubble size represents the impact value, the larger the bubble, the more important the pathway; the bubble color represents the *p* value, the redder the color, the more significant the pathway). The lyophilized powders from the fourth, fifth and sixth cycles from the same reactor were combined, and metabolomic samples were obtained from the combined lyophilized powders of three independent reactors (biological replicates).

The signal communication mechanism of *G. sulfurreducens* cells under electrical signaling disruption was further revealed by the analysis of extracellular differential metabolites and their functional pathways. Since the output current density and electrochemical activity of MFC-TEA gradually increased at Cycles 4, 5, and 6, the samples obtained from these three cycles were representative and combined, and the combined samples were then subjected to metabolomic assays. Based on PLS-DA and parameter settings (VIP value >1.5 and *P* < 0.05), 26 metabolites were significantly upregulated in MFC–TEA compared with MFC-blank (Fig. 5A and Table S2). The differential metabolites were divided into seven categories according to their functions in the KEGG and HMDB databases: (i) antioxidant substances; (ii) primary metabolites; (iii) phospholipid bilayer-related; (iv) amino acid-related; (v) fatty acid-related; (vi) cell signaling-related and (vii) purine- and pyrimidine-related. The presence of antioxidants helps to enhance microbial resistance to unfavorable environments [41], which could be regarded as an adaptable response to TEA-induced electrical signaling disruption. The upregulation of primary metabolites, which are metabolically or physiologically necessary for microbial growth and proliferation, suggested that microbial metabolic processes might be active in MFC-TEA, which was consistent with the gradual increase in output current density of MFC-TEA at Cycles 4, 5, and 6 (Fig. 1A), and also with the results reflected by GO and KEGG analyses (See online supplementary material for a colour version of Fig. S4). In addition, phospholipid bilayer serves as skeletons of cell membrane; amino acids are the building blocks of proteins; fatty acids are involved in biofilm formation and energy storage, and the upregulation of these metabolites further indicated that microbial growth and proliferation might be active.

Notably, the upregulation of cell signaling-related metabolites, especially N-butanoyl-L-homoserine lactone (C4-HSL), suggested that microbes were able to self-secrete endogenous AHL signal molecule to ensure normal cell-to-cell communication under electrical signaling disruption. This was consistent with the results of transcriptomic analysis showing that TEA addition enhanced AHL accumulation by upregulating most AHL-related synthesis genes and downregulating most AHL-related degradation genes (Figs 3A and S5A). Besides, *Geobacter* biofilms have been reported to possess the ability of secreting endogenous AHLs for cell-to-cell communication [8]. Therefore, the detection of C4-HSL confirmed that AHL-mediated quorum sensing was reinforced under electrical signaling disruption. In addition, deoxyguanosine was significantly upregulated (*P* < 0.05), but intracellular c-di-GMP was not detected in the metabolites, mainly because no cell-damaging manipulations were performed during the sample preparation in this experiment. Overall, electrical signaling disruption induced the enhanced synthesis of AHLs signals.

The above differential metabolites were further analysed based on KEGG Pathway database, and the enriched KEGG pathways were ranked in order of importance by combining the results of statistical significance. As shown in Fig. 5B, the important pathways with statistical significance (*P* < 0.05) were Biotin metabolism (k00780), Nicotinate and nicotinamide metabolism (k00760) and Tryptophan metabolism (k00380). Biotin plays important roles in protein synthesis and purine synthesis [42]; nicotinamide can form coenzyme I (nicotinamide adenine dinucleotide, NAD^+^/NADH) and coenzyme II (nicotinamide adenine dinucleotide phosphate, NADP^+^/NADPH), which are closely related to a variety of microbial metabolic processes, such as the generation of high-energy phosphate bonds and lipid metabolism [43]; tryptophan is closely associated with diverse metabolic processes such as carbohydrates, proteins, lipids, vitamins, and trace elements, and has been found to facilitate electron transfer more efficiently than phenylalanine or tyrosine [44]. Therefore, the above analysis further demonstrated that *G. sulfurreducens* cells accelerated extracellular electron transfer efficiency and induced biofilm formation by enhancing the secretion of active substances, amino acid metabolism, fatty acid metabolism, etc.

### Multi-omics analysis explains the compensatory mechanism of chemical communication in response to electrical signaling disruption

The compensatory mechanism between chemical and electrical communication can be established by combining the results of transcriptomic and metabolomic results. Electrical signaling disruption decelerated the formation and electroactivity of *G. sulfurreducens* biofilm, under which the expressions of c-di-GMP-related and quorum sensing-related genes responded adaptively, promoting the accumulation of intracellular c-di-GMP and extracellular AHLs signal molecules (See online supplementary material for a colour version of Fig. S6). Moreover, the detection of C4-HSL in the metabolomics was important evidence that electrical signaling disruption led to the microbes’ own secretion of endogenous AHL signal molecule. Notably, c-di-GMP and AHLs signal molecules could coordinate population-level behaviors and regulate the secretion of EPS, with intracellular c-di-GMP responsive and imperative to adverse external stresses [3, 14, 45], thus constructing a signal communication network responsible for the final biofilm formation.

**Fig. 6:**
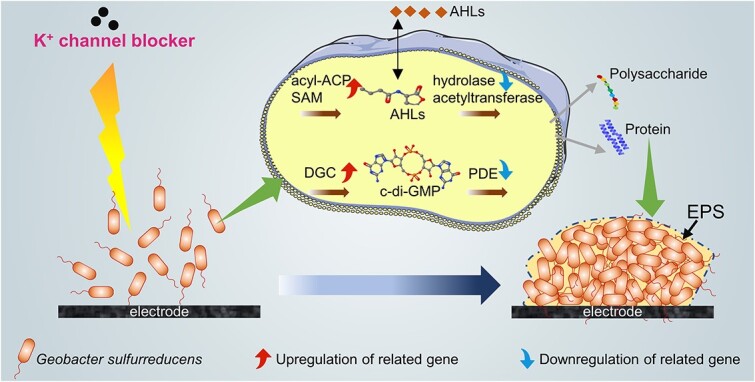
Signal communication mechanism of *G. sulfurreducens* biofilm formation under electrical signaling disruption.

Notably, both prokaryotic transcriptomic and metabolomic enrichment analysis showed that amino acid metabolism, fatty acid metabolism, purine metabolism, pyrimidine metabolism, signal transduction, and membrane transport pathways were upregulated and that microbial growth and proliferation were in an active state, which was consistent with the phenotypic results of a gradual increase in electricity generation performance and biofilm electroactivity at Cycle 4, 5, and 6 (Figs 1 and 2). Therefore, electrical signaling disruption resulted in enhanced chemical communication (including c-di-GMP signaling and AHL-mediated quorum sensing), with a compensatory mechanism among different signaling communications pathways, ultimately inducing the formation of *G. sulfurreducens* biofilm (Fig. 6).

### Implications

To the best of our knowledge, this is the first exploration of the compensatory roles of chemical communication in restoring the formation and electroactivity of *G. sulfurreducens* biofilm in response to electrical communication disruption. The findings showed that *G. sulfurreducens* could enhance c-di-GMP signaling and AHL-mediated quorum sensing to ensure normal cell-to-cell communication under electrical signaling disruption, with intracellular c-di-GMP probably more responsive and imperative to adverse external stresses. Notably, accumulating evidence have demonstrated that bacterial cells utilize intracellular c-di-GMP signaling to respond to diverse stress conditions, regulating the lifestyle transition between the planktonic state and biofilm state. Therefore, the use of c-di-GMP signaling to regulate biofilm formation and stability is intriguing and represents a novel strategy. On one hand, it is proposed that the enhancement of bacterial c-di-GMP signaling would accelerate the formation of electrogenic biofilm, reduce the start-up time of BESs and facilitate the resistance to adverse external conditions, thus contributing to the long-term stability of BESs in practical applications. If the cost is considered, isolating and culturing microbes with unique ability of generating c-di-GMP signals in nature can be taken into account. On the other hand, the inhibition of c-di-GMP signaling by interfering with the production and metabolic processes of c-di-GMP in bacterial cells would greatly benefit the control of the related biological processes, especially for the development of promising anti-biofilm approaches. This would be helpful in the control of biofouling, e.g. the membrane biofouling in membrane bioreactors (MBRs), considering that biofouling could not be completely avoided by simply decreasing extracellular AHLs signals. Therefore, this study sheds light on understanding biofilm formation and its resistance to adverse external stresses from the perspective of bacterial communication, which may suggest a novel strategy for regulating biofilm formation, stability and function using signal communication. In the future, the direct relationship between signal molecules and the properties of the biofilm can be further investigated by monitoring the concentrations of AHLs and c-di-GMP in real time, constructing gene-disrupted mutants, altering bacterial growth environment (with/without TEA) and conducting transcriptomic analysis at different growth cycles of electrogenic biofilms. Besides, further investigations on the interactions among different signal communication pathways in non-bioelectrochemical systems (e.g. other surfaces or even similar nature conditions) should be considered in the future.

## Supplementary Material

Supplementary_data_2024_7_11_ycae096

## Data Availability

All data generated or analysed during this study are included in this published article [and its supplementary information files].

## References

[1] Tian L , Yan X, Wang D et al. Two key *Geobacter* species of wastewater-enriched electroactive biofilm respond differently to electric field. Water Re*s* 2022;213:118185. 10.1016/j.watres.2022.118185 35183018

[2] Zhao N , Liu Y, Zhang Y et al. Pyrogenic carbon facilitated microbial extracellular electron transfer in electrogenic granular sludge via geobattery mechanism. Water Re*s* 2022;220:118618. 10.1016/j.watres.2022.118618 35609427

[3] Guo Y , Liu S, Tang X et al. Role of c-di-GMP in anammox aggregation and systematic analysis of its turnover protein in *Candidatus Jettenia caeni*. Water Re*s* 2017;113:181–90. 10.1016/j.watres.2017.02.018 28214775

[4] Flemming HC , Wingender J, Szewzyk U et al. Biofilms: an emergent form of bacterial life. Nat Rev Microbio*l* 2016;14:563–75. 10.1038/nrmicro.2016.94 27510863

[5] Tan CH , Oh HS, Sheraton VM et al. Convection and the extracellular matrix dictate inter- and intra-biofilm quorum sensing communication in environmental systems. Environ Sci Techno*l* 2020;54:6730–40. 10.1021/acs.est.0c00716 32390423

[6] Prindle A , Liu J, Asally M et al. Ion channels enable electrical communication in bacterial communities. Natur*e* 2015;527:59–63. 10.1038/nature15709 26503040 PMC4890463

[7] Jing X , Chen S, Liu X et al. Potassium channel mediates electroactive biofilm formation via recruiting planktonic *Geobacter* cells. Sci Total Enviro*n* 2022;850:158035. 10.1016/j.scitotenv.2022.158035 35981588

[8] Jing X , Liu X, Deng C et al. Chemical signals stimulate *Geobacter soli* biofilm formation and electroactivity. Biosens Bioelectro*n* 2019;127:1–9. 10.1016/j.bios.2018.11.051 30583280

[9] Das S , Das S, Ghangrekar MM. Bacterial signalling mechanism: An innovative microbial intervention with multifaceted applications in microbial electrochemical technologies: a review. Bioresour Techno*l* 2022;344:126218. 10.1016/j.biortech.2021.126218 34728350

[10] Chen S , Jing X, Tang J et al. Quorum sensing signals enhance the electrochemical activity and energy recovery of mixed-culture electroactive biofilms. Biosens Bioelectro*n* 2017;97:369–76. 10.1016/j.bios.2017.06.024 28624619

[11] Tripathi S , Chandra R, Purchase D et al. Quorum sensing - a promising tool for degradation of industrial waste containing persistent organic pollutants. Environ Pollu*t* 2022;292:118342. 10.1016/j.envpol.2021.118342 34653589

[12] Chattopadhyay I , J RB, Usman TMM et al. Exploring the role of microbial biofilm for industrial effluents treatment. Bioengineere*d* 2022;13:6420–40. 10.1080/21655979.2022.2044250 35227160 PMC8974063

[13] Zhang K , Zhang J, Li J et al. Analyzing the roles of cyclic dimeric guanosine monophosphate (c-di-GMP) on the formation of autotrophic granules and autotrophic biofilm in integrated fixed-film activated sludge (IFAS) reactor. Environ Technol Inn*o* 2022;26:102304. 10.1016/j.eti.2022.102304

[14] Liu X , Cao B, Yang L et al. Biofilm control by interfering with c-di-GMP metabolism and signaling. Biotechnol Ad*v* 2022;56:107915. 10.1016/j.biotechadv.2022.107915 35101567

[15] Humphries J , Xiong L, Liu J et al. Species-independent attraction to biofilms through electrical signaling. Cel*l* 2017;168:200–209.e12. 10.1016/j.cell.2016.12.014 28086091 PMC5497501

[16] Jing X , Yang Y, Ai Z et al. Potassium channel blocker inhibits the formation and electroactivity of *Geobacter* biofilm. Sci Total Enviro*n* 2020;705:135796. 10.1016/j.scitotenv.2019.135796 31806298

[17] Mahto KU , Kumari S, Das S. Unraveling the complex regulatory networks in biofilm formation in bacteria and relevance of biofilms in environmental remediation. Crit Rev Biochem Mol Bio*l* 2022;57:305–32. 10.1080/10409238.2021.2015747 34937434

[18] Waters CM , Lu W, Rabinowitz JD et al. Quorum sensing controls biofilm formation in *Vibrio cholerae* through modulation of cyclic di-GMP levels and repression of *vpsT*. J Bacterio*l* 2008;190:2527–36. 10.1128/JB.01756-07 18223081 PMC2293178

[19] Kim B , Park JS, Choi HY et al. Terrein is an inhibitor of quorum sensing and c-di-GMP in *Pseudomonas aeruginosa*: a connection between quorum sensing and c-di-GMP. Sci Re*p* 2018;8:8617. 10.1038/s41598-018-26974-5 29872101 PMC5988783

[20] Schmid N , Suppiger A, Steiner E et al. High intracellular c-di-GMP levels antagonize quorum sensing and virulence gene expression in *Burkholderia cenocepacia* H111. Microbiolog*y* 2017;163:754–64. 10.1099/mic.0.000452 28463102

[21] Zhang T , Shi XC, Ding R et al. The hidden chemolithoautotrophic metabolism of *Geobacter sulfurreducens* uncovered by adaptation to formate. ISME *J* 2020;14:2078–89. 10.1038/s41396-020-0673-8 32398660 PMC7368069

[22] Zhu Q , Hou H, Wu Y et al. Deciphering the role of extracellular polymeric substances in the regulation of microbial extracellular electron transfer under low concentrations of tetracycline exposure: insights from transcriptomic analysis. Sci Total Enviro*n* 2022;838:156176. 10.1016/j.scitotenv.2022.156176 35613646

[23] Yang G , Huang L, Yu Z et al. Anode potentials regulate *Geobacter* biofilms: new insights from the composition and spatial structure of extracellular polymeric substances. Water Re*s* 2019;159:294–301. 10.1016/j.watres.2019.05.027 31102858

[24] Zhou L , Li T, An J et al. Subminimal inhibitory concentration (sub-MIC) of antibiotic induces electroactive biofilm formation in bioelectrochemical systems. Water Re*s* 2017;125:280–7. 10.1016/j.watres.2017.08.059 28866443

[25] Zhu Q , Qian D, Yuan M et al. Revealing the roles of chemical communication in restoring the formation and electroactivity of electrogenic biofilm under electrical signaling disruption. Water Re*s* 2023;243:120421. 10.1016/j.watres.2023.120421 37523919

[26] Sun D , Cheng S, Wang A et al. Temporal-spatial changes in viabilities and electrochemical properties of anode biofilms. Environ Sci Techno*l* 2015;49:5227–35. 10.1021/acs.est.5b00175 25810405

[27] Xiao Y , Zhao F. Electrochemical roles of extracellular polymeric substances in biofilms. Curr Opin Electroche*m* 2017;4:206–11. 10.1016/j.coelec.2017.09.016

[28] Zhu Q , Hu J, Liu B et al. Potassium channel blocker selectively enriched *Geobacter* from mixed-cultured electroactive biofilm: insights from microbial community, functional prediction and gene expressions. Bioresour Techno*l* 2022;364:128109. 10.1016/j.biortech.2022.128109 36244602

[29] Peng L , Zhang X-T, Yin J et al. *Geobacter sulfurreducens* adapts to low electrode potential for extracellular electron transfer. Electrochim Act*a* 2016;191:743–9. 10.1016/j.electacta.2016.01.033

[30] Okkotsu Y , Little AS, Schurr MJ. The *Pseudomonas aeruginosa* AlgZR two-component system coordinates multiple phenotypes. Front Cell Infect Microbio*l* 2014;4:82. 10.3389/fcimb.2014.00082 24999454 PMC4064291

[31] Bavaharan A , Skilbeck C. Electrical signalling in prokaryotes and its convergence with quorum sensing in *Bacillus*. BioEssay*s* 2022;44:2100193. 10.1002/bies.202100193 35195292

[32] Zhang S , An X, Gong J et al. Molecular response of *Anoxybacillus sp.* PDR2 under azo dye stress: An integrated analysis of proteomics and metabolomics. J Hazard Mate*r* 2022;438:129500. 35792431 10.1016/j.jhazmat.2022.129500

[33] Wang Q , Zhang T, Wu G et al. Deciphering acyl-homoserine lactones-mediated quorum sensing on geotextile bio-clogging in municipal solid waste and bottom ash co-disposal landfills. Waste Mana*g* 2021;124:136–43. 10.1016/j.wasman.2021.02.001 33621757

[34] Sun Y , Guan Y, Zeng D et al. Metagenomics-based interpretation of AHLs-mediated quorum sensing in Anammox biofilm reactors for low-strength wastewater treatment. Chem Eng *J* 2018;344:42–52. 10.1016/j.cej.2018.03.047

[35] Hanzelka BL , Greenberg EP. Quorum sensing in *Vibrio fischeri*: evidence that S-adenosylmethionine is the amino acid substrate for autoinducer synthesis. J Bacterio*l* 1996;178:5291–4. 10.1128/jb.178.17.5291-5294.1996 8752350 PMC178329

[36] Wang Z , Song L, Liu X et al. Bacterial second messenger c-di-GMP: emerging functions in stress resistance. Microbiol Re*s* 2023;268:127302. 10.1016/j.micres.2023.127302 36640720

[37] Liang J , Li W, Zhang H et al. Coaggregation mechanism of pyridine-degrading strains for the acceleration of the aerobic granulation process. Chem Eng *J* 2018;338:176–83. 10.1016/j.cej.2018.01.029

[38] Tan CH , Yeo YP, Hafiz M et al. Functional metagenomic analysis of quorum sensing signaling in a nitrifying community. NPJ Biofilms Microbiome*s* 2021;7:79. 10.1038/s41522-021-00250-3 34711833 PMC8553950

[39] Do EA , Gries CM. Beyond homeostasis: potassium and pathogenesis during bacterial infections. Infect Immu*n* 2021;89:e00766–20. 10.1128/IAI.00766-20 33875474 PMC8486168

[40] Holmes DE , Chaudhuri SK, Nevin KP et al. Microarray and genetic analysis of electron transfer to electrodes in *Geobacter sulfurreducens*. Environ Microbio*l* 2006;8:1805–15. 10.1111/j.1462-2920.2006.01065.x 16958761

[41] Zhang H , Du W, Peralta-Videa JR et al. Metabolomics reveals how cucumber (*Cucumis sativus*) reprograms metabolites to cope with silver ions and silver nanoparticle-induced oxidative stress. Environ Sci Techno*l* 2018;52:8016–26. 10.1021/acs.est.8b02440 29898596

[42] Li R , Li T, Wan Y et al. Efficient decolorization of azo dye wastewater with polyaniline/graphene modified anode in microbial electrochemical systems. J Hazard Mate*r* 2022;421:126740. 10.1016/j.jhazmat.2021.126740 34333409

[43] She J , Sheng R, Qin ZH. Pharmacology and potential implications of nicotinamide adenine dinucleotide precursors. Aging Di*s* 2021;12:1879–97. 10.14336/AD.2021.0523 34881075 PMC8612620

[44] Tan Y , Adhikari RY, Malvankar NS et al. Synthetic biological protein nanowires with high conductivity. Smal*l* 2016;12:4481–5. 10.1002/smll.201601112 27409066

[45] Thibeaux R , Soupe-Gilbert ME, Kainiu M et al. The zoonotic pathogen *Leptospira interrogans* mitigates environmental stress through cyclic-di-GMP-controlled biofilm production. NPJ Biofilms Microbiome*s* 2020;6:24. 10.1038/s41522-020-0134-1 32532998 PMC7293261

