# Supplementary material for: c-di-GMP and AHL signals-triggered chemical communication under electrical signaling disruption restores Geobacter sulfurreducens biofilm formation
Source: ISME Commun. 2024 Jul 20;4(1):ycae096. doi: 10.1093/ismeco/ycae096 (PMC11283642; doi:10.1093/ismeco/ycae096)
Supplement: Supplementary_data_2024_7_11_ycae096 [file supplementary_data_2024_7_11_ycae096.docx]

## Supplementary Information

**c-di-GMP and AHL signals-triggered chemical communication under electrical signaling disruption restores *Geobacter sulfurreducens* biofilm formation**

Qian Zhu ^1, 2^, Yanyan Zheng ^2^, Xingwang Zhou ^2^, Dunjia Wang ^2^, Mengjiao Yuan ^1, 3^, Dingkang Qian ^1, 3^, Sha Liang ^1, 3^, Wenbo Yu ^1, 3^, Jiakuan Yang ^1, 3, 4^, Huijie Hou ^1, 3,^ *, Jingping Hu ^1, 3, 4,^ **

^1^ School of Environmental Science and Engineering, Huazhong University of Science and Technology, 1037 Luoyu Road, Wuhan, Hubei, 430074, China

^2^ College of Chemistry and Chemical Engineering, Hubei Key Laboratory of Pollutant Analysis and Reuse Technology, Hubei Normal University, 11 Cihu Road, Huangshi, Hubei, 435002, China

^3^ Hubei Provincial Engineering Laboratory of Solid Waste Treatment, Disposal and Recycling, 1037 Luoyu Road, Wuhan, Hubei, 430074, China

^4^ State Key Laboratory of Coal Combustion, Huazhong University of Science and Technology, 1037 Luoyu Road, Wuhan, Hubei, 430074, China

***Author for correspondence:**

Huijie Hou, School of Environmental Science and Engineering, Huazhong University of Science and Technology, 1037 Luoyu Road, Wuhan, Hubei, 430074, China. Email: houhuijie@hust.edu.cn.

Jingping Hu, School of Environmental Science and Engineering, Huazhong University of Science and Technology, 1037 Luoyu Road, Wuhan, Hubei, 430074, China. Email: hujp@hust.edu.cn.

**Additional materials and methods**

**Text S1. The details for chromatographic and mass spectrometric conditions** **during untargeted metabolomic analysis.**

The chromatographic conditions were as follows: the column was an ACQUITY UPLC HSS T3 (100 mm × 2.1 mm, i.d., 1.8 μm, Waters, Milford, USA); the mobile phase A was 95% water + 5% acetonitrile (containing 0.1% formic acid), and mobile phase B was 47.5% acetonitrile + 47.5% isopropanol + 5% water (containing 0.1% formic acid); the flow rate was set at 0.40 mL/min; the injection volume was 10 μL. The mass spectrometry conditions were as follows: the scan range was 50 ~ 1000 m/z; the ionization source was electrospray, with temperature of 550 °C; the ion spray voltage floating was −4000 V and 5000 V in negative and positive mode, respectively; the collision energy was set at 40 ± 20 eV. Raw data was processed using Progenesis QI software (Waters Corporation, Milford, USA), and MS/MS fragment spectra were matched to reliable metabolic databases such as KEGG and Human metabolome database (HMDB) to identify metabolites.

Table S1. Transcriptomic expressions of key genes in MFC-TEA *vs*. MFC-blank.

| Gene ID | Gene name | log_2_ (Fold Change) | *p* value |
| --- | --- | --- | --- |
| GS_RS12475 | kdpD | -0.90317 | 0.029229 |
| GS_RS12470 | kdpC | -0.77758 | 0.061684 |
| GS_RS12465 | kdpB | -0.33342 | 0.417917 |
| GS_RS12460 | kdpA | -0.33773 | 0.412331 |
| GS_RS13850 | kefG | -0.14185 | 0.758357 |
| GS_RS13845 | kefB | -0.77635 | 0.065081 |
| GS_RS11630 | GSU2317 | -0.26023 | 0.535479 |
| GS_RS05095 | GSU1016 | -0.3893 | 0.352707 |
| GS_RS02630 | GSU0527 | -0.44876 | 0.283784 |
| GS_RS16765 | GSU3346 | 0.189766 | 0.651647 |
| GS_RS11790 | GSU2350 | -0.3331 | 0.425116 |
| GS_RS06005 | GSU1204 | 0.206891 | 0.620867 |
| GS_RS09460 | metK-1 | 1.254422 | 0.002632 |
| GS_RS04620 | metK-2 | -1.05875 | 0.010829 |
| GS_RS08445 | hom | 1.201222 | 0.004032 |
| GS_RS01210 | acpP-1 | 0.27663 | 0.531131 |
| GS_RS08005 | acpP-2 | 0.782906 | 0.059038 |
| GS_RS09040 | acpS | 0.78672 | 0.088759 |
| GS_RS01440 | fabH-1 | 0.352768 | 0.393803 |
| GS_RS07990 | fabH-2 | 1.058511 | 0.011144 |
| GS_RS07985 | plsX | 0.885596 | 0.03347 |
| GS_RS02380 | GSU0476 | 0.305505 | 0.462915 |
| GS_RS13215 | GSU2626 | -0.49658 | 0.238789 |
| GS_RS01265 | GSU0254 | -0.53965 | 0.243133 |
| GS_RS06440 | GSU1293 | 0.0457 | 0.915037 |
| GS_RS00890 | ato-2 | -1.04371 | 0.011833 |
| GS_RS15655 | GSU3116 | -0.19885 | 0.632887 |
| GS_RS03590 | GSU0719 | -0.90438 | 0.029301 |
| GS_RS06855 | GSU1378 | -1.77748 | 4.10E-05 |
| GS_RS08525 | GSU1708 | -0.26753 | 0.521824 |
| GS_RS09920 | GSU1973 | -1.0812 | 0.013597 |
| GS_RS10285 | GSU2047 | 0.370493 | 0.373329 |
| GS_RS12320 | GSU2450 | -1.24522 | 0.003645 |
| GS_RS15850 | GSU3157 | -0.141 | 0.735759 |
| GS_RS15915 | GSU3171 | -0.419 | 0.317321 |
| GS_RS15975 | GSU3184 | -0.20363 | 0.643078 |
| GS_RS17285 | GSU3451 | -1.41858 | 0.000794 |
| GS_RS09300 | GSU3543 | 1.35385 | 0.002775 |
| GS_RS04490 | GSU0895 | 1.336734 | 0.001435 |
| GS_RS06965 | GSU1400 | 1.998722 | 3.32E-06 |
| GS_RS07755 | GSU1554 | -0.13061 | 0.772894 |
| GS_RS08205 | GSU1643 | 2.789659 | 3.51E-10 |
| GS_RS08265 | GSU1656 | 1.053254 | 0.012635 |
| GS_RS08275 | GSU1658 | 0.027749 | 0.949021 |
| GS_RS08340 | GSU1671 | 2.354413 | 8.21E-08 |
| GS_RS09410 | GSU1870 | 0.554982 | 0.192593 |
| GS_RS10350 | GSU2062 | 0.874962 | 0.036794 |
| GS_RS11610 | GSU2313 | -0.53392 | 0.262911 |
| GS_RS13245 | GSU2632 | 1.157239 | 0.006647 |
| GS_RS14190 | GSU2828 | 0.831269 | 0.052857 |
| GS_RS16810 | GSU3356 | 0.729737 | 0.082174 |
| GS_RS02370 | GSU0474 | -1.13821 | 0.008486 |
| GS_RS02680 | GSU0537 | -2.21703 | 3.01E-07 |
| GS_RS08255 | GSU1654 | 0.410574 | 0.323601 |
| GS_RS05185 | GSU1037 | -1.64777 | 0.00014 |
| GS_RS09695 | GSU1927 | -0.07421 | 0.860492 |
| GS_RS12615 | GSU2511 | -1.93827 | 7.99E-06 |
| GS_RS09755 | GSU1939 | 0.220711 | 0.597027 |
| GS_RS10130 | GSU2016 | 0.779027 | 0.065292 |
| GS_RS03490 | GSU0699 | -1.49208 | 0.000526 |
| GS_RS05050 | GSU1007 | -1.24901 | 0.002727 |
| GS_RS10510 | GSU2094 | -1.22494 | 0.021001 |
| GS_RS12930 | GSU2574 | -0.42695 | 0.31423 |

Table S2. Identified up-regulated differential metabolites in MFC-TEA *vs*. MFC-blank.

| Metabolite | VIP value | *p* value | MFC-TEA | MFC-blank |
| --- | --- | --- | --- | --- |
| Dihydromaleimide beta-D-glucoside | 2.224 | 0.0002554 | 0.8485 | -0.8874 |
| Citronellyl beta-sophoroside | 2.527 | 0.0002737 | 0.8371 | -0.8652 |
| Gentisic acid | 2.0318 | 0.009074 | 0.7014 | -0.808 |
| Pterin | 2.1704 | 0.004465 | 0.9756 | -0.8847 |
| 4-Aminobiphenyl | 2.49 | 0.0002588 | 0.8877 | -0.8831 |
| 1,2,3-Trihydroxybenzene | 1.5821 | 0.03431 | 0.5358 | -0.9404 |
| 5,6-Dimethylbenzimidazole | 1.7083 | 0.01777 | 1.0765 | -0.8081 |
| 2-(Methylamino)benzoic acid | 1.7711 | 0.003811 | 0.8579 | -0.9694 |
| Pentadecanoic acid | 1.575 | 0.007039 | 0.8107 | -0.9964 |
| GPEtn(16:0/16:1) | 1.6508 | 0.0236 | 1.0374 | -0.6634 |
| PE(16:1(5Z)/16:1(5Z)) | 1.9361 | 0.04845 | 1.0606 | -0.531 |
| N-Alpha-acetyllysine | 2.0856 | 0.03517 | 1.1573 | -0.8246 |
| N-Acetylisatin | 2.0669 | 0.0002069 | 0.8658 | -0.8908 |
| 6-Hydroxykynurenic acid | 2.0572 | 0.002539 | 0.8238 | -0.9413 |
| N-Acetyl-DL-methionine | 2.3511 | 0.0005067 | 0.8994 | -0.8849 |
| Ascorbalamic acid | 1.6509 | 0.00121 | 0.8192 | -0.9042 |
| Vinylacetylglycine | 1.9246 | 0.01467 | 1.0604 | -0.8814 |
| N-(2-Hydroxyisobutyl)-2,4,8,10,12-tetradecapentaenamide | 1.5345 | 0.02631 | 1.0513 | -1.0292 |
| N-butanoyl-L-homoserine lactone | 2.1797 | 0.0006662 | 0.9041 | -0.8879 |
| Valyl-Proline | 2.0504 | 0.03354 | 0.5234 | -0.8608 |
| Isoleucylproline | 1.8061 | 0.0003943 | 0.8554 | -0.8985 |
| Gamma-Caprolactone | 1.6068 | 0.02112 | 1.0953 | -0.8105 |
| Methionyl-Histidine | 1.8274 | 0.001642 | 0.927 | -0.8966 |
| Xanthine | 1.5455 | 0.01037 | 0.8843 | -0.6833 |
| Deoxyguanosine | 1.549 | 0.03102 | 0.5365 | -0.8376 |
| 5'-Methylthioadenosine | 1.7003 | 0.007622 | 1.0059 | -0.8959 |


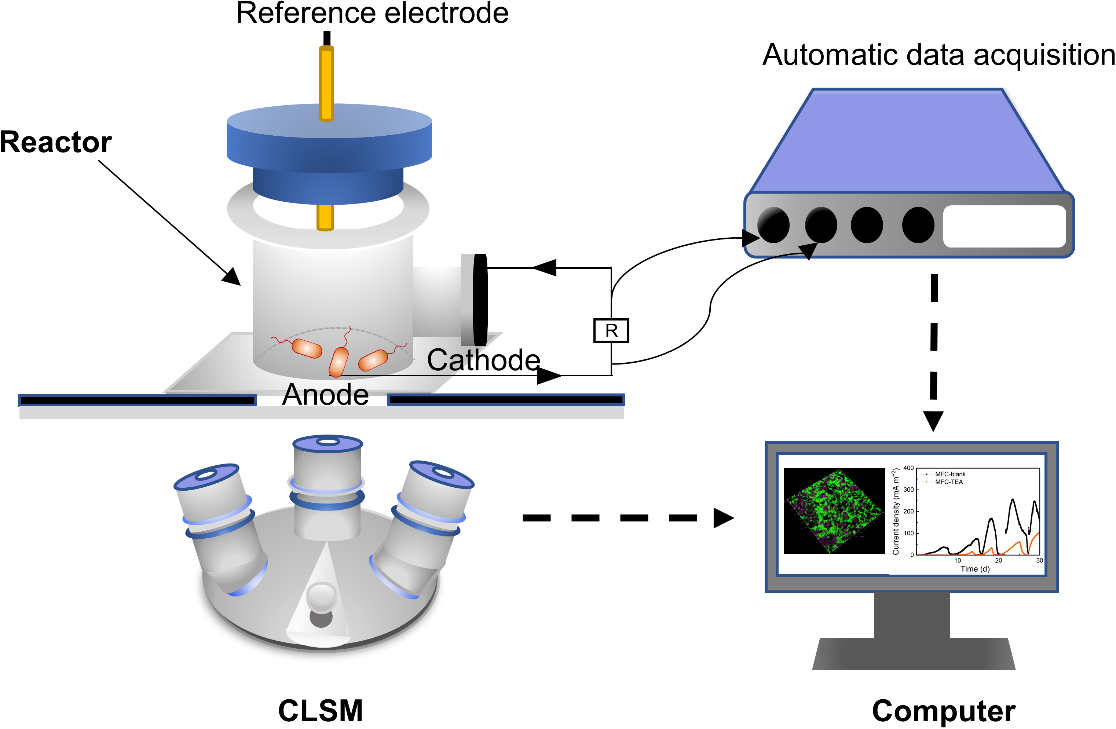


Figure S1. Schematic diagram of an *in-situ* visualization platform, which comprised an *in-situ* MFC system with ITO anode and air-cathode, a confocal laser scanning microscopy (CLSM), a data acquisition system and a computer for data processing.


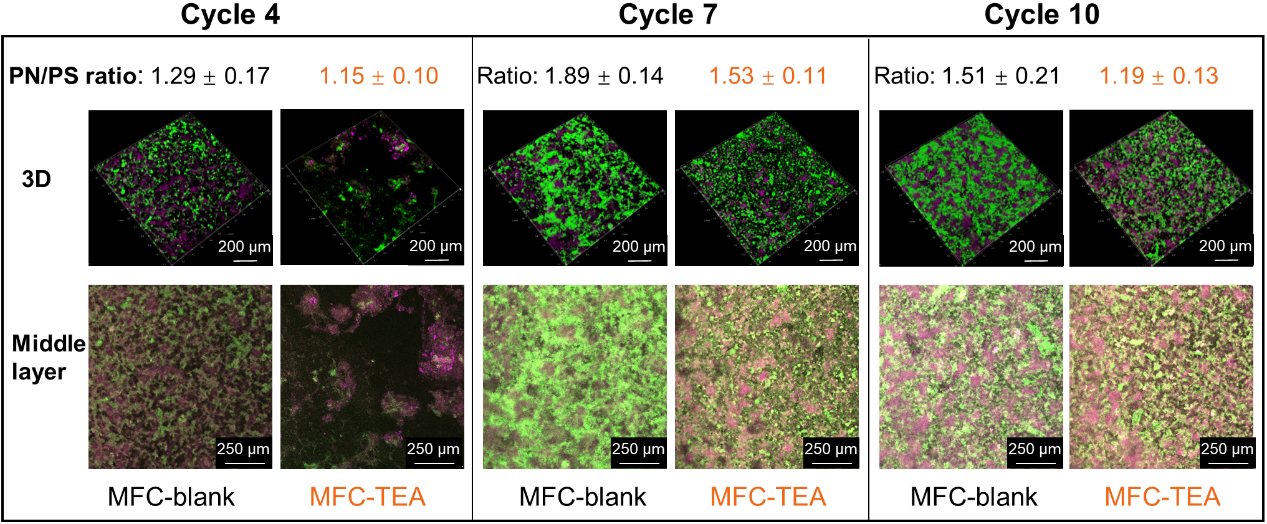


Figure S2. CLSM images of extracellular proteins and polysaccharides in EPS of electrogenic biofilms in MFC-blank and MFC-TEA at Cycle 4, 7 and 10 (PN and PS represent extracellular proteins and polysaccharides, respectively, which were imaged as green and fuchsia, respectively.). The standard deviations of PN/PS ratio were calculated from CLSM images of three independent reactors (biological replicates).


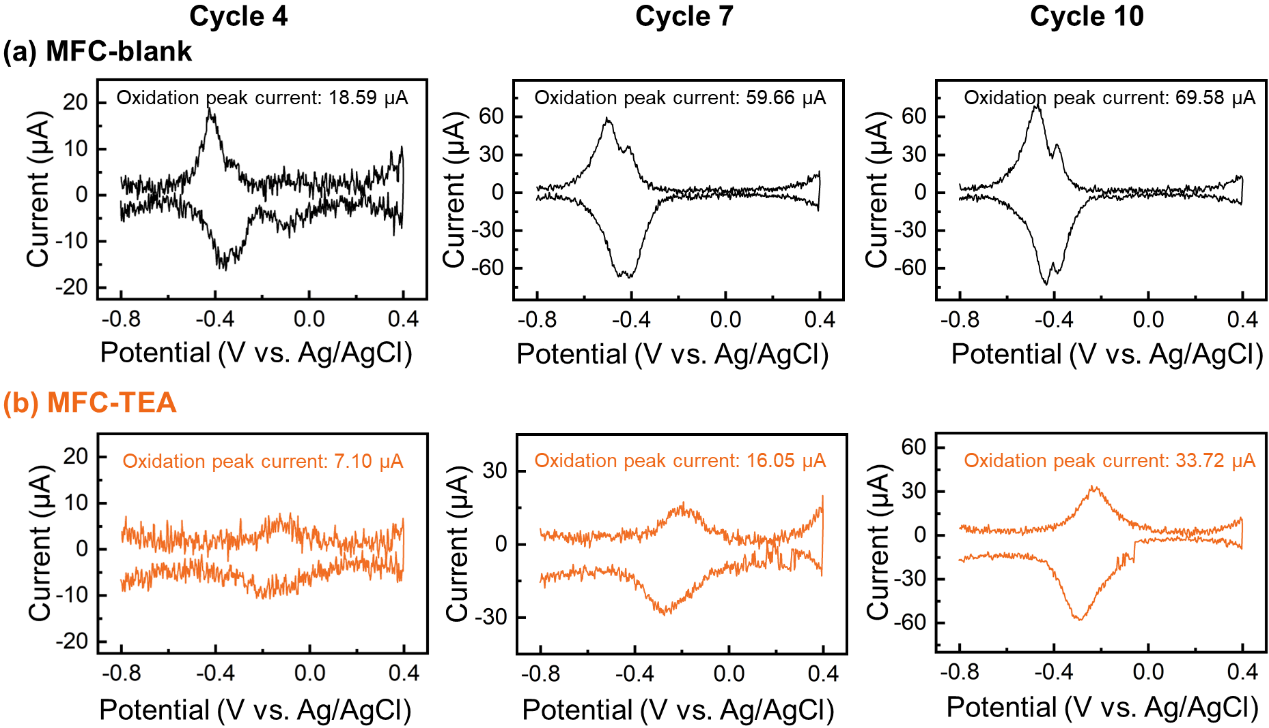


Figure S3. Representative DPV curves of anodic biofilms under non-turnover conditions in (a) MFC-blank and (b) MFC-TEA (the scan range was set at −0.8 ~ 0.4 V, and the pulse height, pulse width, step height and step time were set as 50 mV, 0.3 s, 2 mV and 0.5 s, respectively.).


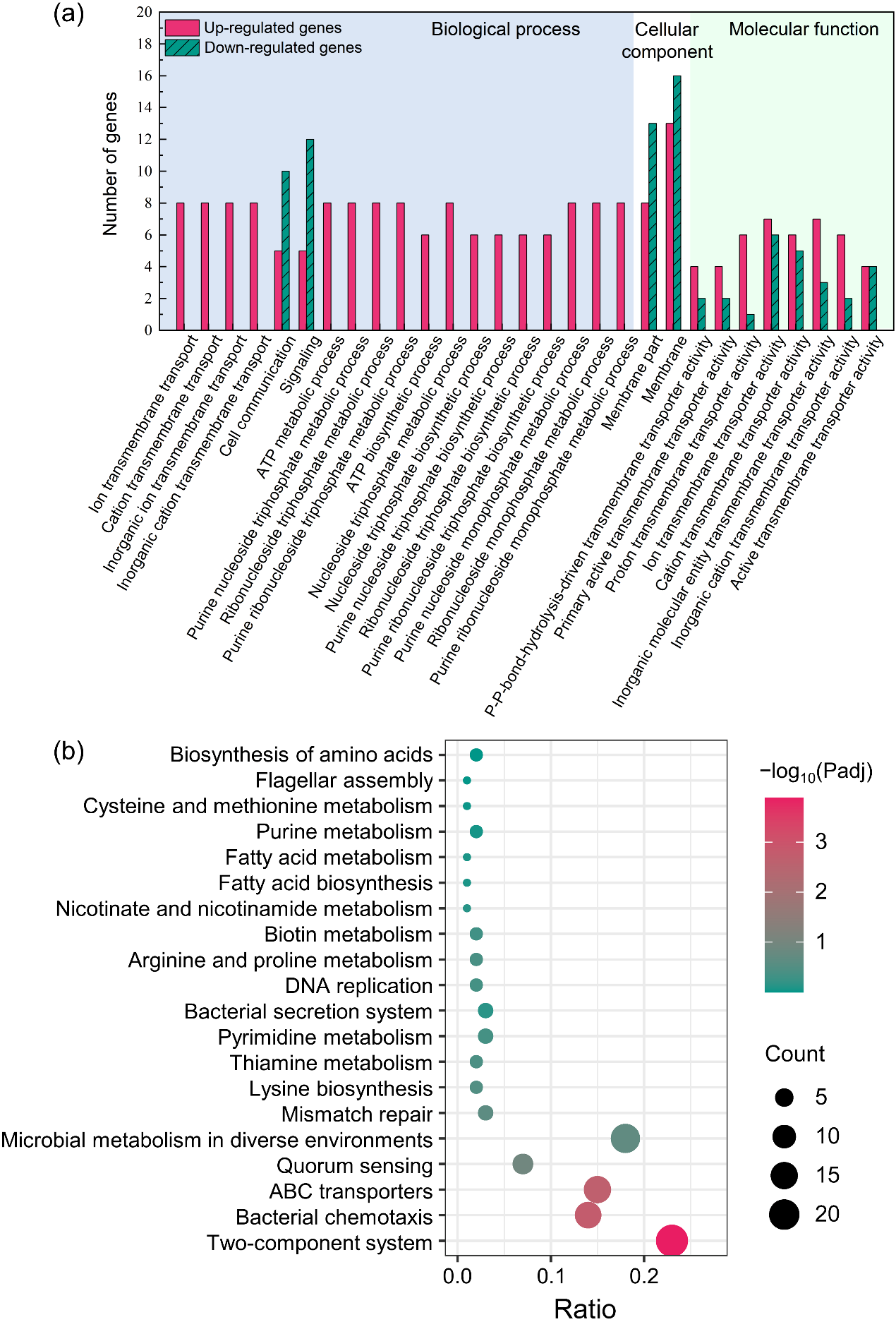


Figure S4. (a) GO enrichment analysis in MFC-TEA *vs*. MFC-blank at the end of cycle 6. (b) Top 20 enriched KEGG pathways in MFC-TEA *vs*. MFC-blank at the end of cycle 6 (Note: the color of circle indicates the Padj of the enrichment analysis, and its diameter is proportional to the number of specific genes that were annotated to the corresponding KEGG pathway). Transcriptomic samples were obtained from the combined anode biofilms of three independent reactors (biological replicates).


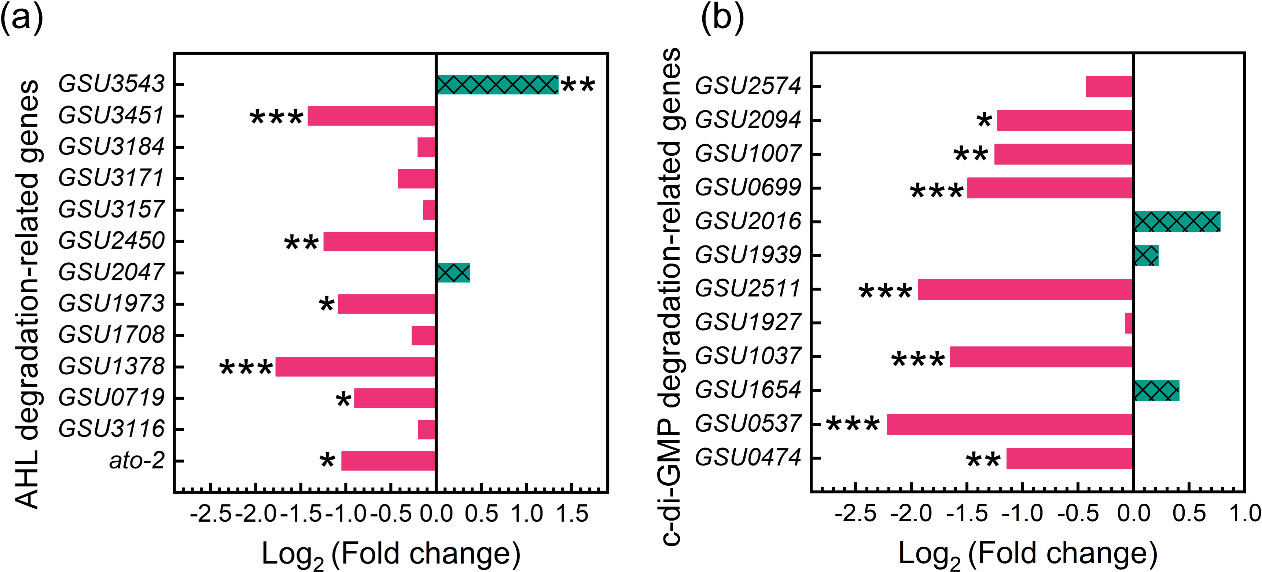


Figure S5. Transcriptomic analysis of functional genes encoding (a) AHL degradation and (b) c-di-GMP degradation. Log_2_ (Fold change) indicates the differential expression values of genes in MFC-TEA *vs*. MFC-blank, and a positive value of Log_2_(Fold change) (green) indicates a higher expression level in MFC-TEA than in MFC-blank, while a negative value (red) denotes a lower expression level in MFC-TEA. Asterisks indicate the statistical significance of difference in MFC-TEA *vs*. MFC-blank, as indicated below: **p* < 0.05, ***p* < 0.01 and ****p* < 0.001. Transcriptomic samples were obtained from the combined anode biofilms of three independent reactors (biological replicates).


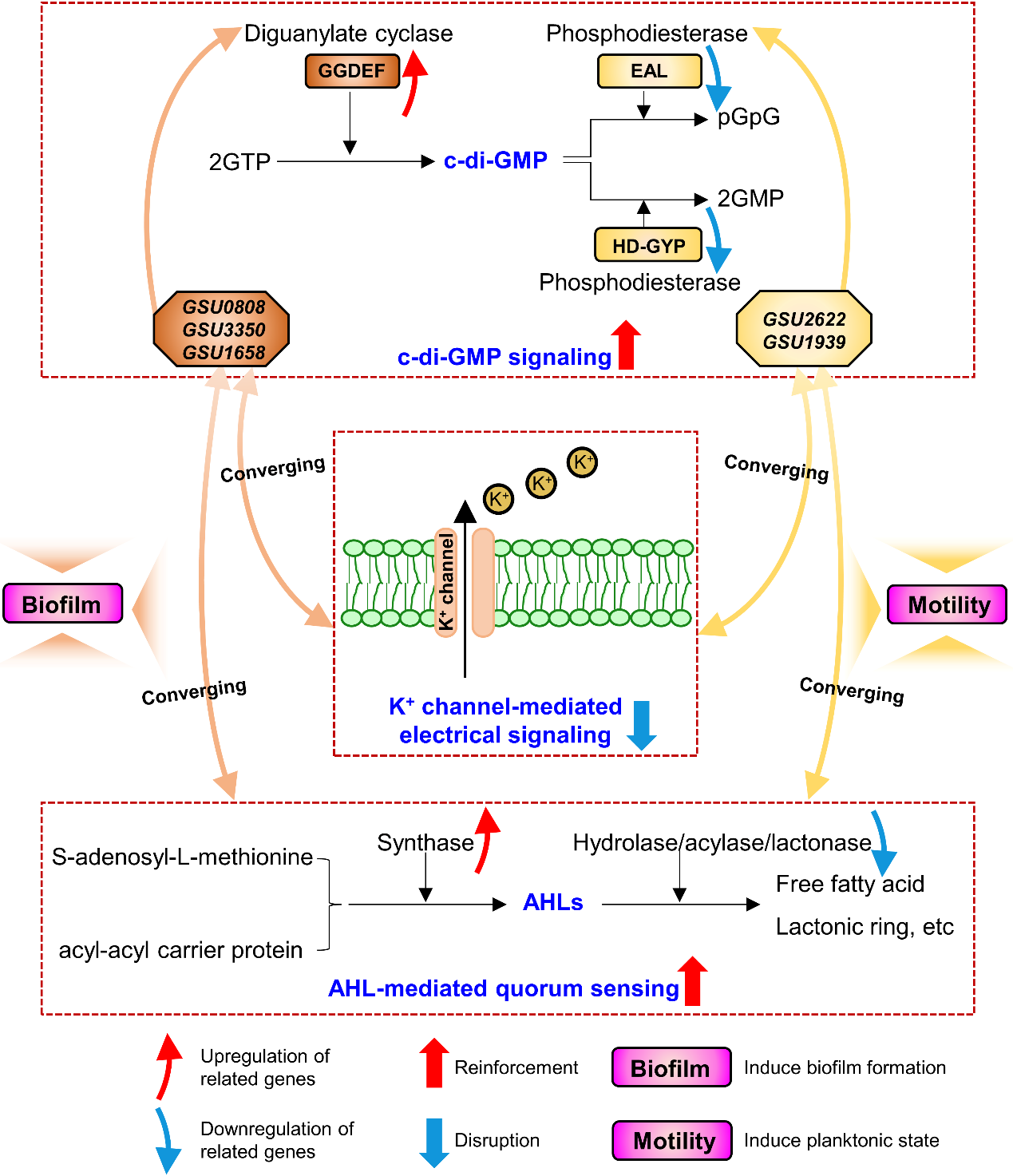


Figure S6. Interactions and convergence among potassium channel-mediated electrical signaling, c-di-GMP signaling and AHL-mediated quorum sensing in *G. sulfurreducens* biofilms: electrical signaling disruption led to enhanced chemical signaling (including c-di-GMP signaling and quorum sensing), and certain genes encoding c-di-GMP synthesis and degradation may play key roles in the convergence of multiple signal communication pathways.
